# Xeno-Free Strategies for Safe Human Mesenchymal Stem/Stromal Cell Expansion: Supplements and Coatings

**DOI:** 10.1155/2017/6597815

**Published:** 2017-10-11

**Authors:** M. Cimino, R. M. Gonçalves, C. C. Barrias, M. C. L. Martins

**Affiliations:** ^1^I3S, Instituto de Investigação e Inovação em Saúde, Universidade do Porto (UP), Porto, Portugal; ^2^Instituto de Engenharia Biomédica (INEB), Universidade do Porto (UP), Porto, Portugal; ^3^Faculdade de Engenharia, Universidade do Porto (UP), Porto, Portugal; ^4^Instituto de Ciências Biomédicas Abel Salazar (ICBAS), Universidade do Porto (UP), Porto, Portugal

## Abstract

Human mesenchymal stem/stromal cells (hMSCs) have generated great interest in regenerative medicine mainly due to their multidifferentiation potential and immunomodulatory role. Although hMSC can be obtained from different tissues, the number of available cells is always low for clinical applications, thus requiring *in vitro* expansion. Most of the current protocols for hMSC expansion make use of fetal bovine serum (FBS) as a nutrient-rich supplement. However, regulatory guidelines encourage novel xeno-free alternatives to define safer and standardized protocols for hMSC expansion that preserve their intrinsic therapeutic potential. Since hMSCs are adherent cells, the attachment surface and cell-adhesive components also play a crucial role on their successful expansion. This review focuses on the advantages/disadvantages of FBS-free media and surfaces/coatings that avoid the use of animal serum, overcoming ethical issues and improving the expansion of hMSC for clinical applications in a safe and reproducible way.

## 1. Introduction

Regenerative medicine aims to repair or replace tissue or organ functions, compromised due to aging, physical damage, congenital defects, or diseases. Cell-based therapies are based on the transplantation of freshly isolated or cultured cells into the site of injury. Those cells are frequently stem cells, which have the ability to self-renew and differentiate along multiple lineage pathways, and thus contribute to tissue repair/regeneration [1].

Among stem cells, human mesenchymal stem/stromal cells (hMSCs) have generated great interest because they are relatively easy to isolate, can be extensively expanded, and present multiple differentiation potential, namely, bone cells (osteocytes), cartilage cells (chondrocytes), and fat cells (adipocytes). Therefore, they are good candidates for cell-based therapeutic approaches towards several kinds of pathologies, such as myocardial infarction [2], graft-versus-host disease [3], Crohn's disease, neurodegenerative and muscle degenerative diseases [4], cartilage and meniscus repair [5], or stroke and spinal cord injury [6]. According to a study from Hart and colleagues, in February 2014 [7], 457 clinical trials involving hMSCs were registered worldwide being China the leader in this ranking. At the time of writing, the number of clinical trials raised until 706 (http://www.clinicaltrials.gov).

Human MSC can be derived from different tissues such as bone marrow (BM-hMSC), adult adipose tissue (AT-hMSC), and mobilized peripheral blood, as well as from placenta and umbilical cord blood (UC-hMSC), being BM the most common source in clinical use. However, hMSC prevalence in all these tissues is low, and the total amounts of isolated cells are insufficient for clinical applications. For example, BM contains approximately 1 in 3.4 × 10^4^ bone cells [8], with total numbers generally decreasing with donor age [9]. The number of required BM-MSCs depends on the type of disease to treat, ranging, for example, from 2 × 10^6^ cells/kg in graft-versus-host disease to 8 × 10^6^ cells/kg in cardiomyopathy and to 10 × 10^6^ cells/kg in respiratory distress syndrome (https://www.clinicaltrials.gov/). Thus, in order to have sufficient cell numbers for successful transplantation, isolated hMSC must be first expanded ex vivo, using effective and safe methods that maintain their key properties in a shorter period of time in order to avoid cell aging and possible contaminations [10].

Several controversies are related with the lack of common standard protocols for hMSC *in vitro* expansion. This is critical since culture conditions may have an impact on the transcriptome, proteome, and cellular organization of hMSCs, which will affect their engraftment and performance upon transplantation [11]. Discrepancy among laboratories includes the choice of basal media and the addition of supplementary factors. Moreover, hMSC being anchorage-dependent cells, culture surfaces are often coated with extracellular matrix (ECM) proteins or other commercially available cell adhesion factors, generating an additional element of discontinuity among expansion protocols. Finally, to reduce variability between preclinical trials, cell culture experiments must comply with good manufacturing practices (GMP) guidelines and every step of cell manipulation must be defined in standard operating procedures (SOP).

In this context, considerable efforts have been made to improve the ex vivo expansion of hMSC for clinical applications, at different levels. This review highlights the disadvantages associated with the use of fetal bovine serum (FBS) as a nutrient-rich medium supplement and focuses on the advantages/disadvantages of different xeno-free and/or serum-free supplements and surfaces/coatings for hMSC expansion.

## 2. Fetal Bovine Serum as a Supplement for hMSC *In Vitro* Expansion

MSC growth *in vitro* must be supported by the addition of a basal media such as Dulbecco's modified Eagle's medium (DMEM), *α*-modified minimum essential medium (*α*-MEM) [12], or media combinations such as 50 : 50 (*v*/*v*) of DMEM : HAM's nutrient mixture F12 [13] which generally include (i) biosynthetic precursors for cell anabolism, (ii) catabolic substrates for energy metabolism, (iii) vitamins and trace elements, and (iv) inorganic ions to maintain the pH and osmolarity of the culture [14]. This basal medium is further supplemented with FBS, a highly rich supplement that contains a cocktail of cell attachment proteins, growth factors, and other important biomolecules.

According to the description by Lalu et al. in 2012 [15], from 36 clinical studies involving hMSC, 27 used FBS as a media supplement and 5 used human serum while 4 did not specify the kind of supplement used.  Table 1 describes some successful clinical trials almost without side effects where hMSCs were expanded in FBS-supplemented medium, even though there are many concerns regarding the use of FBS in terms of scientific ethical and economical disadvantages. These are summarized together with FBS advantages in Table 2.

FBS is an *ill-defined* supplement, with high inconsistency in terms of the quality and quantity of bioactive compounds [16]. Because of the great variability among different FBS batches, preselection of specific lots is often required, which is costly, time consuming, and also hampers comparisons between different research groups [17]. For example, Knepper et al. showed that FBS from three different commercial sources vary on the relative amounts and apparent molecular weights of some transcription factors [18], while Zheng et al. showed that different lots of FBS had varying concentrations of proteins such as growth stimulatory and inhibitory factors, with obvious implications in cell growth rates [19].

When FBS is employed in hMSC expansion for cell therapies, there is also a strong concern regarding contamination with xenogenic compounds and microbiological contaminants, such us viruses, prions, bacteria, fungi, and endotoxins. Directive 2004/23/EC [20] and its recent implementation [21] specify safety measures to be taken into account during donation, procurement, testing, processing, preservation, storage, distribution, and use of human cells and tissues intended for human applications. A notable concern is the potential cross-specific or zoonic transmission of unknown pathogens related to the exposition of cells in culture to animal-derived compounds. Zoonoses bring the risk of virulent diseases such as anthrax, Q fever, and Creutzfeldt-Jakob disease (CJD), where a special concern regards the risk of bovine spongiform encephalopathies (BSE) transmission and its relation to the new variant CJD (vCJD) [22]. Recently, genetic material from bovine viral diarrhea (BVD) virus [23] and new viruses has been detected in bovine serum by massively parallel sequencing [24], a metagenomic technique that can identify viruses and other adventitious agents, without prior knowledge of their nature and being able to reveal either latent or silent infections. Moreover, bovine species of mycoplasma contaminants, often present in FBS, have been frequently isolated from cell cultures [25, 26], but these can be difficult to detect. In fact, batches of contaminated serum may effectively pass the test for mycoplasma as negative samples, as recently reported [27].

Regarding ethical concerns, FBS is harvested from bovine fetuses taken from pregnant cows during slaughter, by cardiac puncture and without any form of anesthesia [28]. During this procedure, fetuses undergo anoxia, a strong oxygen deficiency, and are exposed to pain and/or suffering in a procedure which may be considered ethically inhumane. Finally, the use of FBS in cell culture raises logistic and economic problems: around 1 to 3 fetuses are needed to produce just a liter of serum, implying high costs related to animal feeding, installation, and maintenance of the necessary infrastructures. Recently, an imminent increase of the FBS cost has been notified and justified according to the reduction in supply and the increased demand from life science and pharmaceutical customers. Considering the extensive spread of cell therapy industry, the demand of serum is likely to significantly exceed the amount of maximum worldwide serum availability [29].

## 3. FBS-Free Media Formulations for hMSC *In Vitro* Expansion

Suitable alternatives to FBS for hMSC expansion should ideally guarantee a well-defined composition, a reduced degree of contaminants, low production costs, extended shelf life, and easy availability. Different alternatives have been described in the literature, which can be broadly divided into (1) chemically defined media and (2) media supplemented with human blood derivatives (Table 3).

### 3.1. Chemically Defined Media

Chemically defined (CD) media include only components of known composition. Common strategies for the preparation of CD media have been amply described and validated [13]. In general, formulations for MSC culture consist of a basal media, as already discussed, to which different kinds of supplements are added. Until 15–30 years ago, supplements used in CD media, as highly purified hormones or growth factors, were often obtained from human or animal serum but, nowadays, with the advance of recombinant technologies, it is possible to produce a wide range of human proteins that allow the development of completely xeno-free CD media. Since hMSCs can be isolated from different sources and vary among different donors, the optimal CD media may have different specific requirements concerning medium supplements, which turns the development of a “universal” CD media into a challenging task.

Different CD media (serum free and/or xeno free) for hMSC are nowadays commercially available. For example, TheraPEAK™ MSCGM-CD™ (Lonza) and PowerStem (PAN Biotech GmbH) are able to support hMSC expansion and differentiation; however, CD105 expression may be significantly reduced after culture in those CD media in comparison with serum-supplemented medium [30]. Many of the currently available CD media require precoating of culture substrates with ECM proteins to support cell attachment (Section 4). For example, stemgro hMSC medium (Corning), when used in conjunction with the Corning CellBIND Surface (Section 4.6), enables hMSC attachment and growth comparable to that of serum-containing cultures, including maintenance of MSC multipotency. Another study [31] showed that proliferation of human BM-MSCs was successful on four different commercially available CD xeno-free media (MesenCult-XF, STEMPRO MSC SF, TheraPEAK MSCGM-CD, and Corning stemgro hMSC medium) and in particular upon seeding on a proprietary surface (Corning Synthemax Surface). The Corning medium, associated with the synthetic surface, was able to promote significantly higher long-term cell expansion than traditional serum-containing medium on tissue culture polystyrene (TCPS).

There are however some disadvantages associated with the use of CD media that might limit their wider application in clinical scale hMSC expansion. For example, even if databases of commercially available CD media are accessible online [14], their exact compositions are not specified by the suppliers. Cell-doubling times can vary among different CD media [31], and in some cases, cell morphology and size may differ and alterations in cell vacuoles may arise. Also, the quality/activity of biological compounds in the CD media formulations can generate variability among different batches compromising experiment reproducibility. The use of ECM-like coatings, often mandatory, may also raise some concerns, because of batch-to-batch variability, poor information about their protein content, potential immunogenicity of their components, and time-consuming coating procedure [12]. Often, guidelines for cell adaptation protocols are not provided, which can be a laborious, time-consuming process. Finally, as already stated, this type of media may result in nonoptimal cell growth, as compared to medium supplemented with serum or other animal-derived supplements, as reported in the literature in a study where the performance of different commercially available CD media was compared [32].

### 3.2. Human Blood Derivatives

Several “humanized” supplements derived from human blood have been proposed, namely, (1) autologous or allogeneic human serum (*hS*), (2) human platelet lysate (*hPL*), (3) umbilical cord blood serum (*hUCBS*), and, more recently, (4) industrial GMP human plasma derivatives (supplement for cell culture (*SCC*)). The use of each of these supplements will be described in more detail in the following sections.

#### 3.2.1. Human Serum (hS)

Human serum (hS) can be collected from autologous or allogenic whole blood donations. Autologous hS has been widely described for its positive effects on cell expansion. For example, medium supplemented with 10% *v*/*v* autologous hS was shown to be as efficient as the same amount of FBS for hMSC isolation and expansion, and even better as an inducer of osteogenic differentiation [33]. Yamamoto et al. [34] demonstrated that a patient's autologous hS could be used to expand bone marrow hMSC without compromising their potential for osteogenic differentiation. However, it may be problematic to collect a sufficient amount of autologous serum to propagate hMSC for clinical application. Moreover, depending on the patient, the quality of autologous serum may not be optimal, especially in those undergoing other types of therapies [11]. Also, a negative correlation between donor age and the outgrowth of cells from human trabecular bone was demonstrated in cultures supplemented with autologous serum [35] suggesting that the use of autologous serum is not suitable for elderly patients [36]. To try to overcome these drawbacks, Spees and coworkers demonstrated that, when cells are expanded in medium containing FBS and afterwards transferred to AHS^+^ (autologous human serum supplemented with growth factors), the amount of FBS contaminants can be reduced in 99.99%, which could be used as a strategy to circumvent the low availability of autologous serum [37]. Nevertheless, Martin et al. demonstrated that hMSCs cultured with FBS were contaminated with xeno-derived carbohydrate N-glycolylneuraminic acid (NeuGc) [38, 39]. The subsequent replacement of FBS by hS was insufficient for achieving complete decontamination, since cells express bovine NeuGc on their surface, even after long periods of culture without FBS [40].

On the other hand, hS from allogenic donations can be associated with donor variability. Nevertheless, Witzeneder et al. suggested that pools of 6 donors are sufficient to reduce the variability among human blood samples [41]. However, results from stem cell expansion with allogenic hS still remain controversial, with both positive and negative outcomes described in the literature. For example, autologous serum plus recombinant basic fibroblast growth factor (bFGF) and three recombinant cytokines (Thrombopoietin, Stem Cell Factor, and FL or “Fms-related tyrosine kinase 3 ligand”) have been shown to support expansion of primary marrow stromal cells [42]. Adipose tissue-derived MSC expanded with human allogenic serum pooled from 5 donors exhibited a more spindle-shaped morphology and increased motility than the same cells cultured on FBS, and a significantly higher proliferative rate [43]. Moreover, it has been demonstrated that human serum contains factors that, in comparison to FBS, exert dramatic effects on BM-MSC cell differentiation increasing the osteogenic and adipogenic activity of dexamethasone [44]. However, Anselme et al. [45] observed less formation of fibroblastic colony-forming units (CFU-F) when hMSCs were cultured in hS as compared to FBS-supplemented media. Nevertheless, BM-MSCs cultured in hS before transplantation were more efficient in the production of *in vivo* bone formation than the same cells cultured in FBS [46]. Aldahmash and colleagues [47] did not observe any significant differences in growth rates of an immortalized hMSC line during short-term (10 days) and long-term (100 days) culture in media supplemented with allogenic hS in comparison with FBS. Recently, Choi et al. compared the effects of different concentrations of autologous serum (1, 2, 5, and 10%) on expansion and adipogenic differentiation of AT-MSC using 10% FBS as a control. They found that cell isolation was successful without difference among media, while the proliferation potential follows the trend: 10% autologous serum > 10% FBS = 5% autologous serum > 2% autologous serum = 1% autologous serum [6].

#### 3.2.2. Human Platelet Lysate (hPL)

Human platelet lysate (hPL) can be obtained from blood platelets using different procedures, among which the most common is the freeze/thaw technique developed by Holmqvist and Westermark [48]. This procedure for platelet isolation from blood preserves their *α* granules, which contain a wide range of growth factors such as bFGF, epidermal growth factor (EGF), hepatic growth factor (HGF), insulin growth factor-1 (IGF-1), platelet-derived growth factor (PDGF), transforming growth factor-*β*1 (TGF-*β*1), and vascular endothelial growth factor (VEGF). In comparison to human serum, hPL contains higher levels of strong mitogens (e.g., EGF, PDGF, and TGF-*β*1), whereas the levels of IGF-1 and the protein content are lower due to the removal of immunoglobulins and albumin in the washing procedure [49]. In some studies, hPL has been more efficient than FBS regarding the expansion of hMSC, maintaining their differentiation potential as well as their immunosuppressive properties, namely, in the case of hMSC derived from adipose tissue [50]. Importantly, no difference between the use of freshly prepared and expired platelet concentrates has been observed regarding the proliferation potential and osteogenic differentiation of hMSC [51], suggesting that the bioactivity of this type of supplement is quite stable. The addition of exogenous growth factors has been commonly employed in FBS-supplemented culture to increase cell proliferation rates [52]. However, even if higher proliferation rates have been obtained in cultures supplemented with FBS combined with bFGF, as compared to FBS only, those rates remained lower than the ones obtained in medium supplemented with 10% hPL [53]. Interestingly, hPL without the addition of anticoagulant forms a soft hydrogel, providing a three-dimensional (3D) scaffold, which has been described to increase *in vitro* expansion of hMSC by mimicking a more natural 3D context than the commonly used 2D polystyrene surfaces [50]. Finally, platelet derivatives have been already tested in clinical trials with good results for the treatment of tendinopathies and osteoarthritis [54, 55] and in hearth failure [56].

However, the use of hPL also presents some disadvantages. First, it is a supplement for cell culture that, similarly to FBS, is not precisely defined. A high variability exists between individual hPLs, as their composition is strongly dependent on donor-related factors, such us age, gender, blood group, and platelet individual counts [57]. Nevertheless, pooling different donations can reduce variability. Schallmoser et al. demonstrated that a pool of 15 donations can improve standardization [58]. Also, hPL isolation process may affect platelet degranulation and, consequently, growth factor content of hPLs. Additional variability may result from the filtering procedure, storage typology, or the content in heparin or anticoagulants [55]. Moreover, as observed for other human-derived media supplements, there is a concern regarding the possibility of immunological reactions, in allogenic settings, and transmission of human diseases. However, Castiglia et al. [59] recently described a method named *ihPL* (inactivated hPL), which ensures efficient generation of safe hPL batches, by virus inactivation through UVA light.

#### 3.2.3. Human Umbilical Cord Blood Serum (hUCBS)

Human umbilical cord blood serum (hUCBS) can be easily obtained following normal delivery, thus normally screened for the most common bacterial or viral contamination, and its use does not raise any ethical issues. It contains high levels of soluble growth factors and more than 60 proteins, namely, albumin, transferrin, and fibronectin (FN) in high concentrations, with different roles in cell growth and stem cell differentiation [12]. Phadnis et al. [60] demonstrated that BM-hMSC displayed a 32-fold increase in cell number 5 days after seeding in UCBS against a 10-fold increase in FBS culture conditions. Moreover, 10% UCBS has been proved to be more efficient in promoting hMSC osteogenic differentiation than 10% FBS because of its enhancement of osteocalcin promoter expression [61].

In general, UCBS can be considered advantageous as a cell culture supplement considering the easy availability of this allogeneic source, the easy and inexpensive isolation procedure [62], and the near absence of contaminants. On the other hand, some limitations must be considered, such as the high lot-to-lot variability, associated to donor-related features and the presence of adventitious agents that may eventually escape routine screening procedures.

#### 3.2.4. Industrial GMP Human Plasma Derivatives (Supplement for Cell Culture (SCC))

A new type of supplement cell culture (SCC) has been recently described for *in vitro* cell expansion [63], consisting on a GMP pharmaceutical grade xeno-free human plasma-derived supplement. SCC is obtained from human plasma through cold-ethanol industrial fractionation [64]. Once plasma is collected from healthy donors at USA-based FDA-licensed plasmapheresis centers, plasma pools from over 1000 different donors are assembled in a single plasma unit. Every individual donation is tested for viral markers, and all plasma is tested, using nucleic acid techniques, for the presence of DNA or RNA from relevant pathogenic agents, in agreement with the European and American legislations. In addition, SCC production procedure includes a specific viral inactivation step (gamma irradiation) besides other purification steps with additional pathogen removal capacity [63].

SCC has already been successfully used as a supplement for *in vitro* cell culture to basal medium that supports culture of stem cells such as human embryonic stem cells (hES), induced pluripotent stem cells (iPSC) derived from human dermal fibroblast [65], and hMSCs [63]. When hMSCs were expanded in SCC (15% SCC reconstituted in basal medium (DMEM : HAM's nutrient mixture F12)) containing a cocktail of growth factors and other elements such as insulin, sodium selenite, and ethanolamine, cell yield was similar to the one obtained for hMSC expanded in commercial medium obtained from hMSC suppliers (Lonza Group and PromoCell). Moreover, SCC-supplemented medium maintained hMSC in an undifferentiated state during expansion and kept their capacity of adipogenic, chondrogenic, and osteogenic differentiation, under inducing conditions [63].

However, SCC being a supplement in very recent development, only few data are available in the literature regarding its potential use as an hMSC medium supplement. Thus, further investigations will help to a better characterization and improve additional applications. Its introduction in the market as other alternatives to FBS will show its potentiality as a supplement for *in vitro* cell expansion.

## 4. Surfaces and Coatings to Promote hMSC Attachment and Growth

Anchorage-dependent cells, such as hMSCs, are supported *in vivo* by the ECM, an assembly of many proteins and glycosaminoglycans (GAGs) that provides a 3D environment for their organization into tissues. Specific interactions between cell surface receptors and ligands on the ECM mediate intracellular signaling pathways and control gene expression, cytoskeletal organization, and cell morphology that are involved in key cellular activities such as cell adhesion, migration, proliferation, and differentiation [66].

When cells are cultured with FBS-supplemented medium, a protein layer readily adsorbs to the tissue culture polystyrene (TCPS) surface, the material generally used in the commercial cell culture flasks. This protein layer contains several adhesive proteins, such as fibronectin (FN) and vitronectin (VN) that are essential to mediate early stages of cell attachment [67], before cells are able to produce their own ECM [68]. In contrast, hMSC cultured in the absence of FBS does not properly attach to TCPS [69]. A common strategy for hMSC expansion in serum-free conditions is their preincubation in FBS as a transient adaptation step to serum-free medium [69]. This process can confer to the cells the necessary elements for attachment. However, this is not acceptable in a GMP setting, demonstrating the need of identifying defined cell attachment factors and also designing new culture surfaces that support hMSC adhesion and proliferation, while maintaining its multipotency, in the absence of FBS.

### 4.1. Surface Coating with Cell-Adhesive Proteins

The simplest strategy to improve the attachment of hMSC in culture cell consists in precoating TCPS surfaces with purified adhesive proteins that can be isolated from human plasma or synthesized by recombinant technologies. Surface modification with a FN coating is probably the most widely used strategy for improving hMSC adhesion and proliferation in serum-free conditions. FN is a multifunctional, ECM glycoprotein that contains several functionally and structurally distinct domains, including cell-binding domains such as the RGD (Arg-Gly-Asp) sequence that are recognized by cell surface integrins, namely, *α*5*β*1 and *α*v*β*3 [70]. Other coating proteins, such as laminin (LN), collagen type I (COL I) and collagen type IV (COL IV), and gelatin, also showed to improve hMSC adhesion and growth in serum-free medium [69]. In contrast, the number of attached hMSC on poly-D-lysine precoated surfaces was very low even when studies were performed in the presence of FBS [69]. Moreover, cells did not show their typical fibroblast-like spindled-shape morphology, which could be related with their low spreading capacity in this type of coating [71]. Ode et al. also reported that TCPS precoated with FN as well as COL I, II, III, and IV and fibrinogen (FG), but not with LN, induces hMSC adhesion and proliferation in serum-reduced conditions (0.1% *v*/*v* FBS) [72]. LN, an ECM protein especially abundant within the basal lamina of epithelial and endothelial tissues, has been described as an important mediator of cell attachment and migration [73]. Yet, the effect of LN-coated surfaces on hMSC adhesion and growth is not clear since opposite outcomes have been reported [69, 72, 74, 75]. Differences in hMSC behavior on LN-coated surfaces could be related with (i) protein conformation, which is dependent on the type of substrate, LN concentration, and incubation time; (ii) type of LN, as different forms have been identified; and (iii) disparities in terms of cell culture protocols, including the type of medium used.

Ogura et al. [76] also demonstrated that hMSC attachment and spreading on FN-coated dishes were increased when compared to albumin-coated dishes. This is not surprising since albumin, the protein that exists in higher concentration in serum and the first to reach and adsorb to most of the surfaces, is known by its nonadhesive characteristics [77].

Besides affecting hMSC attachment and proliferation, ECM protein coatings can also influence cell differentiation into specific lineages. For example, it is known that FN plays a pivotal role in hMSC osteogenic differentiation, while inhibiting adipogenesis [78]. VN and COL I coatings were also shown to induce MSC osteogenic differentiation [74, 78] through interaction with specific COL I receptor *α*_1_*β*_1_ and VN receptor *α*_V_*β*_3_ integrins. This fact was observed even in the absence of soluble osteogenic inducers [74]. LN-1 and LN-5 were shown to promote hMSC osteogenic differentiation in xeno-free conditions (human plasma and platelet extract) [79], but LN-5 suppress chondrogenic differentiation [75]. Since surface chemistry will influence the amount and conformation of the adsorbed protein layer and thus the exposure of specific integrin-binding sequences, the type of substrate used will affect hMSC differentiation profile even when the same protein coating was used [80].

TCPS coated with keratins, isolated from human hair, was recently suggested as an alternative to other most common adhesive proteins for hMSC *in vitro* expansion [81]. This protein contains the LDV (Leu-Asp-Val) cell adhesion motif recognized by cell membrane integrin *α*_4_*β*_1_. Moreover, human hair keratins can be easily obtained in abundance even from an autologous source. However, more studies need to be performed in order to further validate its suitability for this kind of applications.

### 4.2. Surface Modifications to Promote Adequate Adsorption of Adhesive Proteins

The type of underlying substrate used for cell culture also influences hMSC adhesion and proliferation, since it dictates the nature, conformation, and orientation of adsorbed proteins and, consequently, the exposure of active ligands (cell-binding domains) for cell recognition and binding. Commercial cell culture flasks are commonly made of TCPS. These surfaces are obtained by modifying hydrophobic polystyrene surfaces, often by gas-plasma treatment, to turn them hydrophilic and negatively charged. This promotes the adsorption of serum attachment proteins such as FN and VN, in relation to other proteins such as albumin [82], providing better surfaces for subsequent cell adhesion.

In fact, it is currently well known that cell-binding domains of FN are hidden when the protein is in its native structure, becoming exposed upon adsorption on surfaces with adequate wettability and charge [83]. So, the effect of adsorbed FN on cell attachment largely depends on the properties of the underlying substrate. For example, Dolatshahi-Pirouz et al. demonstrated that although FN adsorption was higher on gold (Au) surfaces than on hydroxyapatite (HA) surfaces, the morphology of attached hMSC was more regular on HA. This was explained by the higher exposure of cell-binding domains on FN adsorbed on HA, which was confirmed using monoclonal antibodies directed against FN cell-binding domains [84]. The influence of surface wettability (hydrophilic versus hydrophobic surfaces) on selective adsorption of FN and VN from FBS and respective exposure of cell-binding domains was also studied using self-assembled monolayers (SAMs) prepared by mixing different ratios of OH- (hydrophilic-) and CH_3_- (hydrophobic-) terminated alkanethiols [67]. hMSC adhesion and proliferation increased with the increase of surface hydrophilicity (increase of OH/CH_3_) which was directly correlated with the increased exposure of cell-binding domains on adsorbed FN and VN [67]. However, Curran et al. [85] did not find differences in hMSC adhesion and viability among −CH_3_, −OH, −NH_2_, −SH, and −COOH glass silane-modified surfaces in the presence of FBS. Nevertheless, they demonstrated that surface chemistry was able to modulate hMSC potential of differentiation. They reported that only −CH_3_ surfaces were able to maintain MSC phenotype, since cells were expanded without losing their differentiation skills under appropriated stimulus. NH_2_ and −SH surfaces promoted osteogenesis differentiation while −OH and −COOH surfaces support chondrogenic differentiation even in basal conditions [85].

Although all these studies were performed in the presence of FBS, they highlight the importance of surface chemistry on the amount and conformation of adsorbed adhesive proteins, an essential step in the design of substrates for serum-free or xeno-free hMSC cultures.

### 4.3. Surface Chemical Modifications with Cell-Adhesive Peptides

Since the process of surface coating by simple protein adsorption does not provide a high level of control over the presentation of cell-binding domains, different strategies have been idealized to covalently immobilize cell-binding motifs (e.g., RGD) on a substrate [86, 87]. With this strategy, problems associated with surface coatings with proteins from animal or human origin can be overcome, since cells may directly bind to the functionalized substrate. Other advantages of chemically modified synthetic surfaces are their higher stability during storage and their low batch-to-batch variability.

An array with different RGD densities in a bioinert background was developed using different concentrations of the immobilized peptide sequence RGDSP (Arg-Gly-Asp-Ser-pro) on ethylene glycol-terminated SAMs. Results demonstrated that higher peptide densities induce higher MSC spreading and focal adhesion formation in the presence of FBS [88]. In fact, it is well known that although surfaces functionalized with RGD motifs can increase hMSC attachment and spreading, its efficacy strongly depends on its surface density [87] and patterning [89]. Surfaces functionalized with RGDs for xeno-free hMSC culture are already commercialized by BD Biosciences (Section 4.6).

### 4.4. Surface Coatings with Decellularized ECM

Single-protein coatings as the ones described above lack the complexity of cell-secreted ECM. Coatings with decellularized ECM can be used as an alternative to single proteins to improve cell growth and proliferation *in vitro* without the loss of their stem cell properties [90]. To obtain these coatings, hMSCs are cultured on tissue culture plates (or other substrate of interest) for enough time to allow cells to produce their own ECM, after which the coating is decellularized through appropriate processing, leaving only the ECM components. The decellularized ECM coating can be maintained in the original substrate, where subsequent hMSC populations can be directly cultured or can be collected and transferred to other substrate without losing its instructive potential [90].

Lai et al. described the characteristics of an ECM coating produced by hMSC before and after decellularization. Using confocal microscopy, it was possible to visualize the localization of COL I and III, FN, small leucine-rich proteoglycans such as biglycan and decorin, and major components of basement membrane such as the large molecular weight proteoglycan perlecan and LN [91].

A coating with ECM derived from human fetal MSC (fMSC) improved adult hMSC proliferation maintaining their multipotency, in comparison with ECM derived from adult MSC and TCPS. This fact could be related with the higher proliferation capacity of fMSC with associated higher amounts of ECM production [92]. These results are very promising for ex vivo expansion of hMSCs; however, they were not performed in xeno-free conditions. Moreover, the available amount of fMSCs associated with ethical issues could be a concern for this strategy.

The most commonly used decellularization processes are based on the detachment of intact cells using enzymatic processes or the lysis of cells using detergent compounds. However, all these processes have disadvantages, namely, the use of proteases such as trypsin, which can damage the ECM components, and the lysis of cells that can contaminate the resultant ECM with intracellular components. Rao Pattabhi et al. described a new decellularization protocol based on cold EDTA to remove intact cells from ECM without enzymes and detergents and with minimal ECM damage and contamination. They demonstrated that ECM derived from hMSCs obtained using this process enhances the proliferation of naive hMSC maintaining their potential for osteogenesis and adipogenesis [93].

However, all these assays were performed in standard medium containing FBS since their aim is the development of protocols that can enhance hMSC proliferation maintaining their multipotency. Thus, additional experiments are needed to adapt these strategies to xeno-free MSC cultures.

### 4.5. Surface Modification with GAGs

GAGs are ECM polysaccharides that are involved in a variety of extracellular and intracellular functions and are being widely explored for tissue engineering applications, when used both as surface coatings [94] and as 3D scaffolds for hMSC culture [95, 96].

Heparin is a linear GAG containing several sulphate and carboxyl groups, with a high negative charge density and with several well-characterized binding domains to different growth factors and ECM proteins, such as FGF-2, FN, VN, LN, COL, and bone morphogenetic proteins (BMP) [97, 98].

Heparin-functionalized surfaces may be engineered to attract and bind soluble growth factors, concentrating them on the surface, which avoids the need of using extra high concentrations of soluble growth factors during *in vitro* hMSC expansion. However, besides promoting hMSC adhesion and proliferation, these surfaces also induce osteogenic differentiation in standard culture medium [95]. This fact was associated with heparin ability to sequester from the culture medium not only proteins essential for hMSC adhesion (e.g., FN and VN) and proliferation (e.g., FGF2) but also proteins involved in osteogenic differentiation (e.g., BMPs) [99].

In a different approach, surfaces can be engineered to specifically sequester serum-borne heparin to cell-material interface and thus to attract and bind soluble growth factors. Heparin-binding surfaces were prepared by covalent immobilization of a small heparin-binding peptide (GGGKRTGQYKL) and the integrin-binding peptide (RGDSP) on bioinert SAMs. RGDSP was included to improve cell adhesion to bioinert SAMs. In the presence of FBS, these surfaces were able to enhance hMSC proliferation and osteogenic differentiation by amplifying the activity of endogenous FGF-2 and BMP, respectively [99].

However, it was also suggested that the upregulation of hMSC osteogenic differentiation could be related with heparin-induced alterations on FN conformation. Recent evidences pointed out that heparin induces a more extended conformation of fibrillar FN included in ECM scaffolds. Extended FN may expose hidden binding sites for many growth factors (e.g., FGF-2, BMP-2, and VEGF) that are fundamental on hMSC osteogenic differentiation [100].

### 4.6. Commercial Coatings and Modified Surfaces for MSC Culture

Different xeno-free protein-based coatings or modified culture surfaces for hMSC culture have been developed and are currently commercially available. Their use is nowadays widespread for hMSC *in vitro* expansion, especially when in combination with serum-free or chemically defined media. Some examples are described in Table 4, which is intended to be illustrative rather than all inclusive.

## 5. Conclusions

With impressive biological properties, hMSCs are nowadays considered one of the most promising cell types for cellular therapies, already demonstrated in many clinical trials using hMSC. Nevertheless, hMSCs are scarce and to achieve sufficient numbers for cell transplantation, an *in vitro* expansion step is required. This procedure requires improvements in order to guarantee a safe preparation of hMSC as therapeutic products.

Specific challenges concern the substitution of animal components such as FBS during hMSC *in vitro* expansion, since its uses raises scientific, economic, and ethical issues. To improve clinical use of hMSC for advanced therapies, cell culture/expansion under xeno-free conditions should be encouraged. Current strategies include the replacement of FBS with chemically defined media or human plasma derivatives such as hS, hUCBS, hPLs, and a more recent GMP-compliant supplement for cell culture (SCC). Beyond soluble components, recent investigations consist also in the development of refined culture surfaces for hMSC *in vitro* culture: in order to augment the adsorption of adhesive and/or growth-promoting agents, surfaces can be functionalized with adequate chemical groups and modified by immobilization of peptide ligands or whole ECM proteins such as GAGs and FN. However, most of these studies are being conducted in the presence of serum and additional experiments are needed to transpose these strategies to xeno-free MSC cultures.

In recent years, different promising experimental settings have been proposed towards clinical safety of hMSC ex vivo expansion and illustrated herein. However, there is still a lack of standardized protocols for this intent; thus, high-quality translation research and exchange among the numerous research groups interested in the field are demanded.

## Figures and Tables

**Table 1:** Successful clinical trials involving hMSC expanded *in vitro* with FBS-containing medium.

Type of disease	Treatment	Reference
Luminal Crohn's disease	Allogenic hMSCs	Forbes 2014 [101] (^∗^)
Ischemic stroke	Autologous MSCs	Lee 2010 [102] (^∗∗^)
Stroke injury	Autologous MSCs	Bang 2005 [103]
Amyotrophic lateral sclerosis	Intraspinal cord implantation	Mazzini 2008 [104]
Grade IV acute graft-versus-host disease	Haploidentical MSC	Le Blanc 2004 [105]
Brest cancer	Autologous MSCs	Koc 2000 [106]
Acute myocardial infarction	Autologous MSCs	Chen 2004 [107]

^∗^Australia being a country with no incidence of BSE and vCJD, the use of certified FBS for hMSC ex vivo expansion is still accepted; ^∗∗^no adverse events in terms of zoonoses transmission were observed; however, since the vCJD latency period may last many years, long-term follow-up is needed.

**Table 2:** FBS use advantages and drawbacks.

Use of FBS as a supplement for cell culture	
Advantages	Disadvantages
Furnishes a cocktail of growth factors required for *in vitro* cell growth	Ill defined
Universal: suitable for all cell types	Lot-to-lot variability
	Possible contamination of the cell surface with xenogenic compounds that may influence cell behavior
	Possible microbiological contamination (virus, prions bacteria, endotoxins, and fungi)
	Economical: worldwide availability
	Ethical problems: requires the painful death of bovine fetuses

**Table 3:** Xeno-free supplements for cell culture.

Human blood derivatives				Not human origin
Human serum (HS)	Human platelet lysate (hPL)	Human umbilical cord serum (hUCS)	Human plasma fractions (supplement for cell culture (SCC))	Chemically defined media (CD)
*Advantages*				
No ethical problems	No ethical problems	No ethical problems	No ethical problems	No ethical problems
No risk of xenogenic compound transmission	No risk of xenogenic compound transmission	No risk of xenogenic compound transmission	No risk of xenogenic compound transmission	No risk of xenogenic compound transmission
Easy and inexpensive procedure	Contains high level of GF	Easy and inexpensive procedure	GMP compliant: compatible with pharmaceutical grade advanced therapies	No risk of transmission of human diseases
		Contains high level of GF and proteins (transferrin, albumin, and fibronectin)	Little batch to batch variability (derived from an industrial plasma pool constituted from over 1000 donations)	
		Easy-available allogenic source	Provides cell growth and attachment factors	
			Lyophilized: convenient for transport and storage	

*Disadvantages*				
Variability between individual donations	Variability between individual donations	Variability between individual donations	Ongoing: further investigation will help to a better characterization	Cell specific
Possibility of transmission of human diseases	Possibility of transmission of human diseases	Possibility of transmission of human diseases		Ill-defined because it lacks of information provided by the companies
Low availability in terms of amount of donation	Low availability in terms of amount of donation	Low availability in terms of amount of donation		Development may be expensive and time consuming
Discrepancy of results in allogenic settings	Not well defined composition in terms of GF content			Cell-adaptation may be necessary
Quality in autologous settings may not be ideal	Quite laborious and expensive procedure			
Often requires the addition of cytokines and GF				

**Table 4:** Commercially available coating strategies for hMSC *in vitro* culture.

Name	Company	Composition	Validation (cell types)
*Soluble coatings*			
CELLstart™	Thermo Fisher	Not disclosed	hMSC, human embryonic stem cells (hESC), and human neural stem cells (hNSC)
Nutristem MSC attachment solution	Biological industries	Affinity-purified human plasma fibronectin	BM-hMSC, AT-hMSC, UC-hMSC
Xuri MSC attachment solution	GE Healthcare	Affinity-purified human plasma fibronectin	Validated for human BM-hMSC, AT-hMSC, and UC-hMSC

*Modified plasticware*			
BD PureCoat™ ECM Mimetic Cultureware: collagen and fibronectin	Corning	Synthetic animal-free peptides covalently linked to a proprietary surface: (i) fibronectin-derived RGD peptide (ii) collagen I-derived peptide	BM-hMSC and AT-hMSC human umbilical cord blood-derived MSC (isolation)
Corning® Synthemax® Surface	Corning	Peptide acrylate coating functionalized with a vitronectin-derived peptide	Validated for hPSCs and other adult stem cell types
